# Efficacy of Admission Screening for Extended-Spectrum Beta-Lactamase Producing *Enterobacteriaceae*

**DOI:** 10.1371/journal.pone.0062678

**Published:** 2013-04-26

**Authors:** Christopher F. Lowe, Kevin Katz, Allison J. McGeer, Matthew P. Muller, for the Toronto ESBL Working Group

**Affiliations:** 1 Department of Laboratory Medicine and Pathobiology, University of Toronto, Toronto, Ontario, Canada; 2 Deparment of Infection Prevention and Control, North York General Hospital, Toronto, Ontario, Canada; 3 Department of Microbiology, Mount Sinai Hospital, Toronto, Ontario, Canada; 4 Division of Infectious Diseases, St. Michael’s Hospital, Toronto, Ontario, Canada; 5 Department of Medicine, University of Toronto, Toronto, Ontario, Canada; Amphia Ziekenhuis, The Netherlands

## Abstract

**Objective:**

We hypothesized that admission screening for extended-spectrum β-lactamase-producing *Enterobacteriaceae* (ESBL-E) reduces the incidence of hospital-acquired ESBL-E clinical isolates.

**Design:**

Retrospective cohort study.

**Setting:**

12 hospitals (6 screening and 6 non-screening) in Toronto, Canada.

**Patients:**

All adult inpatients with an ESBL-E positive culture collected from 2005–2009.

**Methods:**

Cases were defined as hospital-onset (HO) or community-onset (CO) if cultures were positive after or before 72 hours. Efficacy of screening in reducing HO-ESBL-E incidence was assessed with a negative binomial model adjusting for study year and CO-ESBL-E incidence. The accuracy of the HO-ESBL-E definition was assessed by re-classifying HO-ESBL-E cases as confirmed nosocomial (negative admission screen), probable nosocomial (no admission screen) or not nosocomial (positive admission screen) using data from the screening hospitals.

**Results:**

There were 2,088 ESBL-E positive patients and incidence of ESBL-E rose from 0.11 to 0.42 per 1,000 inpatient days between 2005 and 2009. CO-ESBL-E incidence was similar at screening and non-screening hospitals but screening hospitals had a lower incidence of HO-ESBL-E in all years. In the negative binomial model, screening was associated with a 49.1% reduction in HO-ESBL-E (p<0.001). A similar reduction was seen in the incidence of HO-ESBL-E bacteremia. When HO-ESBL-E cases were re-classified based on their admission screen result, 46.5% were positive on admission, 32.5% were confirmed as nosocomial and 21.0% were probable nosocomial cases.

**Conclusions:**

Admission screening for ESBL-E is associated with a reduced incidence of HO-ESBL-E. Controlled, prospective studies of admission screening for ESBL-E should be a priority.

## Introduction

The proliferation of antibiotic resistant organisms, in particular multi-drug resistant Gram negative organisms, is an emerging public health crisis [1]. Extended-spectrum β-lactamase-producing *Enterobacteriaceae* (ESBL-E) are a major contributor to this problem due to their increasing incidence, multi-drug resistance and increasing carbapenem use, potentially contributing to the emergence of carbapenem resistant *Enterobacteriaceae* (CRE) [1]–[4].

Despite the importance of this problem, there is little consensus and no definitive guidelines on the optimal infection control interventions to reduce the transmission of endemic ESBL-E in hospitals [5]. The use of admission screening to detect ESBL-E colonized patients is particularly controversial, due to its substantial associated costs [6], the potentially harmful effects of patient isolation [7], and the lack of empiric evidence demonstrating its efficacy in reducing the incidence of nosocomial ESBL-E transmission in the endemic setting [5]. Despite this, admission screening is an effective component of infection control interventions to control ESBL-E (and CRE) outbreaks [8]–[11] and is recommended in several guidelines for the control of drug resistant Gram positive organisms such as methicillin resistant *Staphylococcus aureus* (MRSA) and vancomycin resistant Enterococci (VRE) [12].

We recently conducted a survey of hospitals in Toronto, Canada to ascertain their approaches to infection control for both ESBL-E and CRE. One finding of our study was that approximately 50% of hospitals used admission screening to identify patients with ESBL-E [13]. This variation in practice provided an opportunity to evaluate the effectiveness of this intervention.

## Methods

### Ethics Statement

This study was approved by Research Ethics Boards at St. Michael’s Hospital, Mount Sinai Hospital, Sunnybrook Health Sciences Centre, University Health Network, North York General Hospital, Toronto East General Hospital, Lakeridge Health, Southlake Regional Health Centre, and The Scarborough Hospital and the Credit Valley Hospital Research Review Committee. Written consent by patients was not obtained since this was a retrospective study, the data was analyzed anonymously and patient care was not affected by the study.

### Study Design, Study Setting and Population

We conducted a retrospective cohort study of ESBL-E incidence at 6 screening and 6 non-screening hospitals in Toronto, Canada. Data on all clinical and screening specimens positive for ESBL-E were collected from 2005 to 2009. Of the 6 hospitals performing admission screening, all used admission rectal swabs. Four of the 6 hospitals (R1–R4) conducted risk-factor based screening while 2 conducted universal screening (R5, R6). The demographic characteristics and infection control practices employed for the control of ESBL-E at all 12 hospitals are presented in Table 1. All 4 risk-factor based screening programs included travel to an endemic country as a risk factor. Other risk factors used included previous ESBL-E colonization/infection (R1), previous hospitalization (R1,R4), transfer from a long-term care facility (R4), admission to a specific ward (intensive care unit (R1,R2,R3,R4), neonatal intensive care unit (R1,R4), general medicine (R1) or general surgery (R1)) or increased risk of environmental contamination such as diarrhea or draining wound (R2,R3). Screening practices were in place at all screening hospitals prior to the start of the study period. All screening strategies were combined together for the analysis of screening hospitals as the total screens per 1,000 admissions were comparable. No non-screening hospitals had previously had an admission screening program. Practices were also stable at the non-screening hospitals.

**Table 1:** Characteristics of participating hospitals.

	Non-Screening Hospital (N = 6)	Screening Hospital (N = 6)	p-value
**Hospital Characteristics**			
Inpatient Days per Month:			
Mean	11,842	9,205	0.19
Median	11,437	9,327	
Range	7,369–14,627	5,220–12,492	
Mean Admissions per Year:			
Mean	19,605	18,567	0.61
Median	21,270	18,055	
Range	9,882–26,317	13,188–25,875	
Mean Number of Beds:			
Mean	395	376	0.64
Median	421	382	
Range	260–460	227–510	
Number of ESBL-E Clinical Isolates	1,357	731	0.02
Number of ESBL-E Bloodstream Infections	175	77	0.06
Number of Positive ESBL-E Rectal Screens	N/A	1,887	
**Infection Control Practices**			
Contact Precautions for all identified ESBL-E†	50.0% (3/6)	66.6% (4/6)	1.0
Private Room	33.3% (2/6)	66.6% (4/6)	0.57
Cohort	83.3% (5/6)	33.3% (2/6)	0.24
Precautions implemented for the duration of admission*	50.0% (3/6)	16.6% (1/6)	0.55
ESBL-E flagged in electronic clinical database	33.3% (2/6)	66.6% (4/6)	0.57
**Clinical Isolates Positive for ESBL- Producing Organism**			
*Escherichia coli*	80.3% (1,131)	87.4% (639)	0.91
*Klebsiella pneumoniae*	14.8% (209)	8.1% (59)	0.23
*Klebsiella oxytoca*	1.2% (17)	4.5% (33)	0.23
**Culture Site**			
Urine	72.6% (985)	75.6% (553)	0.54
Blood	12.9% (175)	10.5% (77)	0.44
Respiratory	6.0% (81)	4.5% (33)	0.41
Wound	4.1% (56)	5.9% (43)	0.27
Abscess	3.3% (45)	1.8% (13)	0.06
Other	1.1% (15)	1.6% (12)	0.58

† The remaining hospitals used contact precautions for patients considered at risk of transmission of ESBL (e.g. with diarrhea, incontinence or uncontained wound drainage).

* The other 3 non-screening hospitals discontinued precautions after 1 negative specimen from the original site (1 hospital) or after 3 negative screening samples each separated by 1 week (2 hospitals). For the other 5 screening hospitals, 3 discontinued after 3 negative screening samples each separated by 1 week and 2 had no written protocols established for discontinuation of precautions.

Infection control strategies were similar at the 12 hospitals, although use of a private room for ESBL-E colonized/infected patients was more common in screening hospitals (4/6 vs. 2/6) (Table 1). Two screening and 3 non-screening hospitals used contact precautions only for ESBL-E patients at increased risk of transmission (e.g. incontinent or draining wound). Contact precautions included the use of gowns and gloves prior to entry into an ESBL-E positive room. Patients were placed in precautions only after they were identified as ESBL-E positive by screen or clinical culture. Control screening (i.e. screening of patients for ESBL-E carriage on a regular schedule, not including screening on admission) was not performed at any hospital. There was variation with respect to the duration patients remained in precautions, from discontinuation after 1 negative culture to 3 negative cultures separated each by 1 week to continuation for the entire admission (Table 1).

### Microbiology Methods and Inclusion/Exclusion Criteria

Adult inpatients from all clinical services with a clinical culture and/or an admission rectal screen positive for an Ambler Class A ESBL-producing *Escherichia coli*, *Klebsiella pneumoniae* and *Klebsiella oxytoca* were included. Only the first clinical isolate and/or admission screen for each patient was included.

All hospitals used similar standard methods for identification of ESBL-E positive isolates. Admission screens were plated onto MacConkey cefpodoxime (2 µg/mL) agar. Clinical isolates intermediate or resistant to a 3^rd^ generation cephalosporin (cefpodoxime, ceftriaxone or ceftazidime) or colonies with growth on the MacConkey cefpodoxime agar were confirmed as ESBL-producers with the double disk diffusion test (ceftriaxone, ceftazidime and aztreonam plus/minus clavulanic acid and cefoxitin) [14].

### Study Outcomes and Definitions

The primary outcome for this study was the incidence of hospital-onset (HO) ESBL-E per 1,000 inpatient days. Patients were considered to have HO-ESBL-E if an ESBL-E was identified from a clinical specimen obtained >72 hours after admission without any prior clinical cultures yielding ESBL-E. Patients were considered to have community-onset (CO) ESBL-E if clinical cultures were positive within 72 hours of hospital admission. Admission screening specimens were not used to classify cases as HO-ESBL-E or CO-ESBL-E for the primary analysis, as such specimens were collected only at the screening hospitals and their inclusion would result in a bias in favour of the screening hospitals.

However, to confirm the accuracy of these definitions, we re-classified all HO-ESBL-E cases identified at the screening hospitals based on the results of their admission screen. Specifically, HO-ESBL-E cases were re-classified as confirmed nosocomial cases if the admission screen was negative, probable nosocomial cases if the admission screen was not performed and non-nosocomial (or colonized at admission) cases if the admission screen was positive.

Secondary outcomes included the incidence of HO-ESBL-E stratified by organism (e.g. *E. coli* or *K. pneumoniae*), the incidence of HO-ESBL-E bacteremia and the ratio of hospital-onset to community-onset cases (HO/CO ratio). This ratio provides a crude estimate of the number of nosocomial transmissions that resulted per ESBL-positive patient admitted. For the screening hospitals, we also evaluated the undetected ratio which is the ratio of patients identified only via admission screening compared to all patients identified as ESBL-E positive via admission and/or clinical cultures. The undetected ratio estimates the proportion of patients that would not be identified as ESBL-E colonized in hospitals without an admission screening program [10].

### Statistical Analysis

Data were exported from the laboratory informatics system of all hospitals, merged and stored in a Microsoft Excel 2007 (Redmond, Washington) database. Analysis was conducted using SAS Version 9.2 (Cary, North Carolina). Data in Table 1 were analyzed with Fisher’s exact test and Student’s t-test, where appropriate. Crude case numbers at all facilities were adjusted for inpatient days and incidence rates presented as cases per 1,000 inpatient days.

For the primary analysis comparing the incidence of HO-ESBL-E between screening and non-screening hospitals, a negative binomial model was developed with the number of HO-ESBL-E cases per hospital as the outcome, offset by the natural logarithm of 1,000 inpatient days. The impact of screening strategy was evaluated after adjustment for the year of isolate collection and the baseline incidence of CO-ESBL-E at each hospital. Similar methods were used to compare the incidence of HO-ESBL-E bacteremia between screening and non-screening hospitals, and to compare results independently for each organism.

## Results

### Comparison of Screening and Non-Screening Hospitals

**Figure 1:**
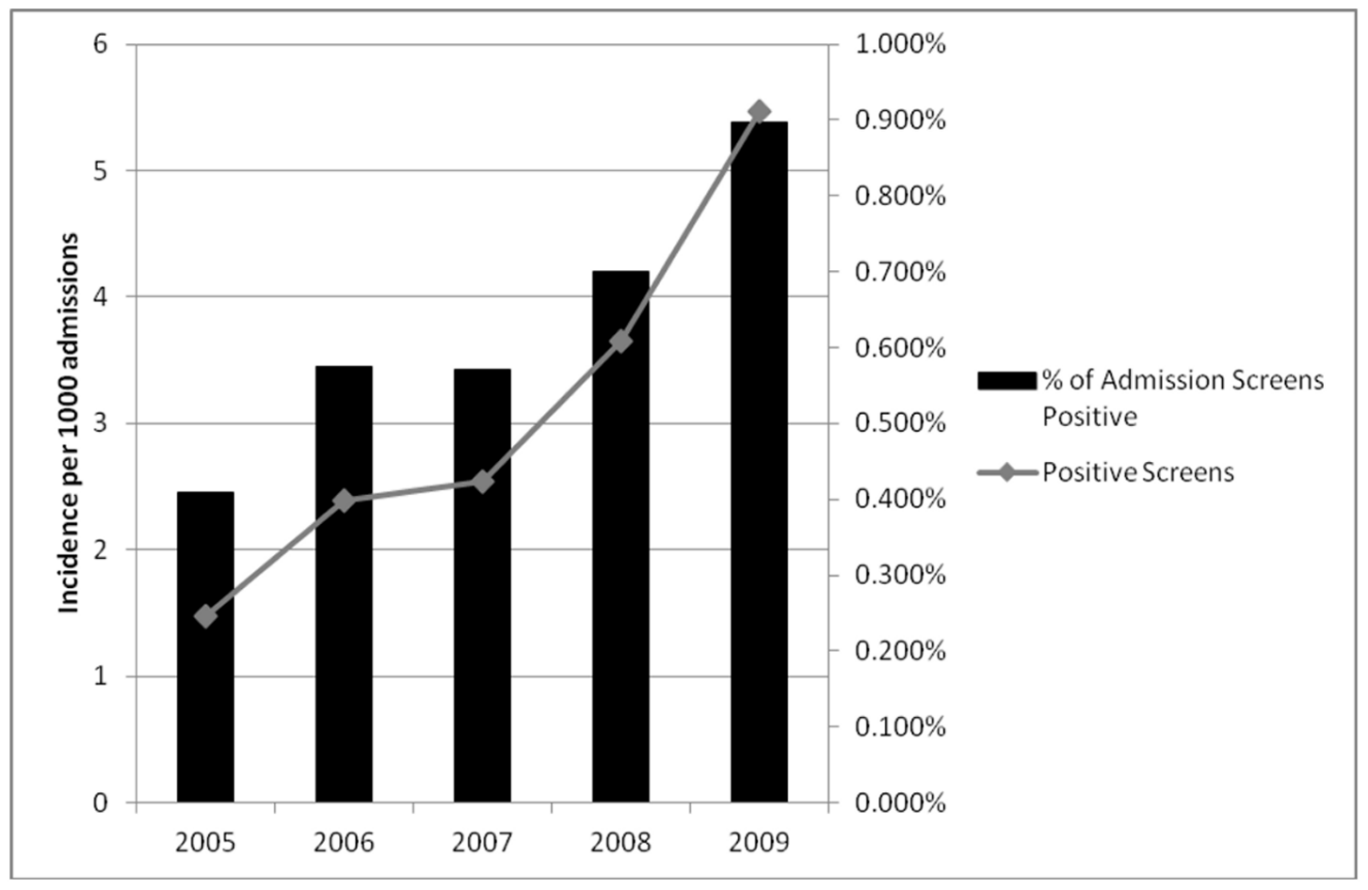
Incidence of positive admission screens and percent positivity of rectal screening for ESBL-producing Enterobacteriaceae*. *Only hospitals R1, R5 and R6 were included as the total number of screens for the remaining hospitals was incomplete.

Over the 5 year study, there were 3,975 patients admitted with an ESBL-E positive clinical culture (2,088) or admission rectal screen (1,887). Table 1 describes the characteristics of screening and non-screening hospitals. The overall incidence of all ESBL-E positive clinical isolates rose from 0.11 cases per 1,000 inpatient days in 2005 to 0.42 cases per 1,000 inpatient days in 2009. The median rate of admission screening at the 6 screening hospitals was 550 per 1,000 admissions (range: 266–872). Both the absolute number of positive admission screens for ESBL-E and the proportion of screens that were positive increased over the 5 year study period (Figure 1). At the 6 screening hospitals, total positive screens per year rose from 101 (5.4 per 1,000 admissions) in 2005 to 660 (35.5 per 1,000 admissions) in 2009.

**Figure 2:**
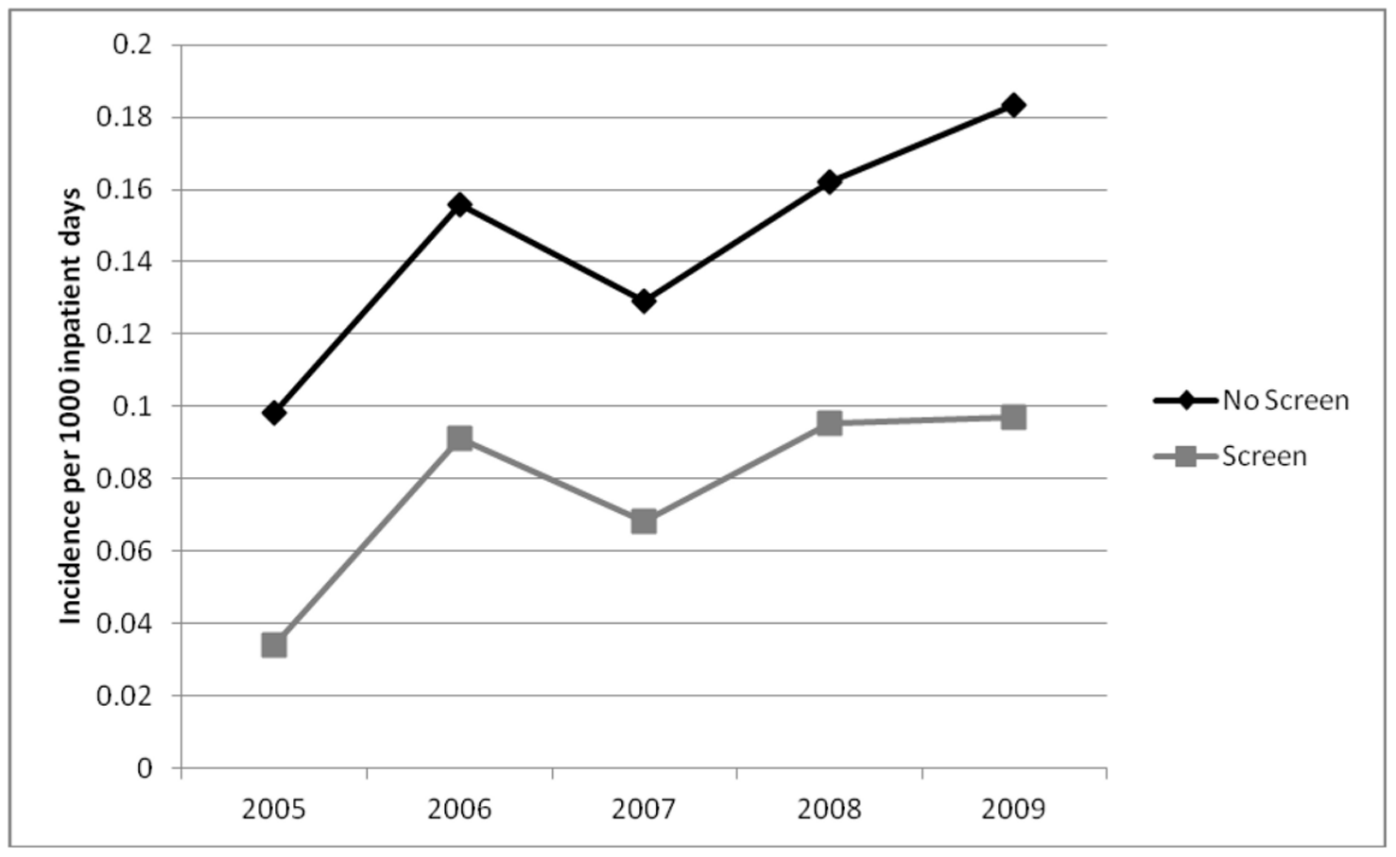
Incidence of hospital-onset cases of ESBL-producing *Enterobacteriaceae* in non-screening compared to screening hospitals.

**Figure 3:**
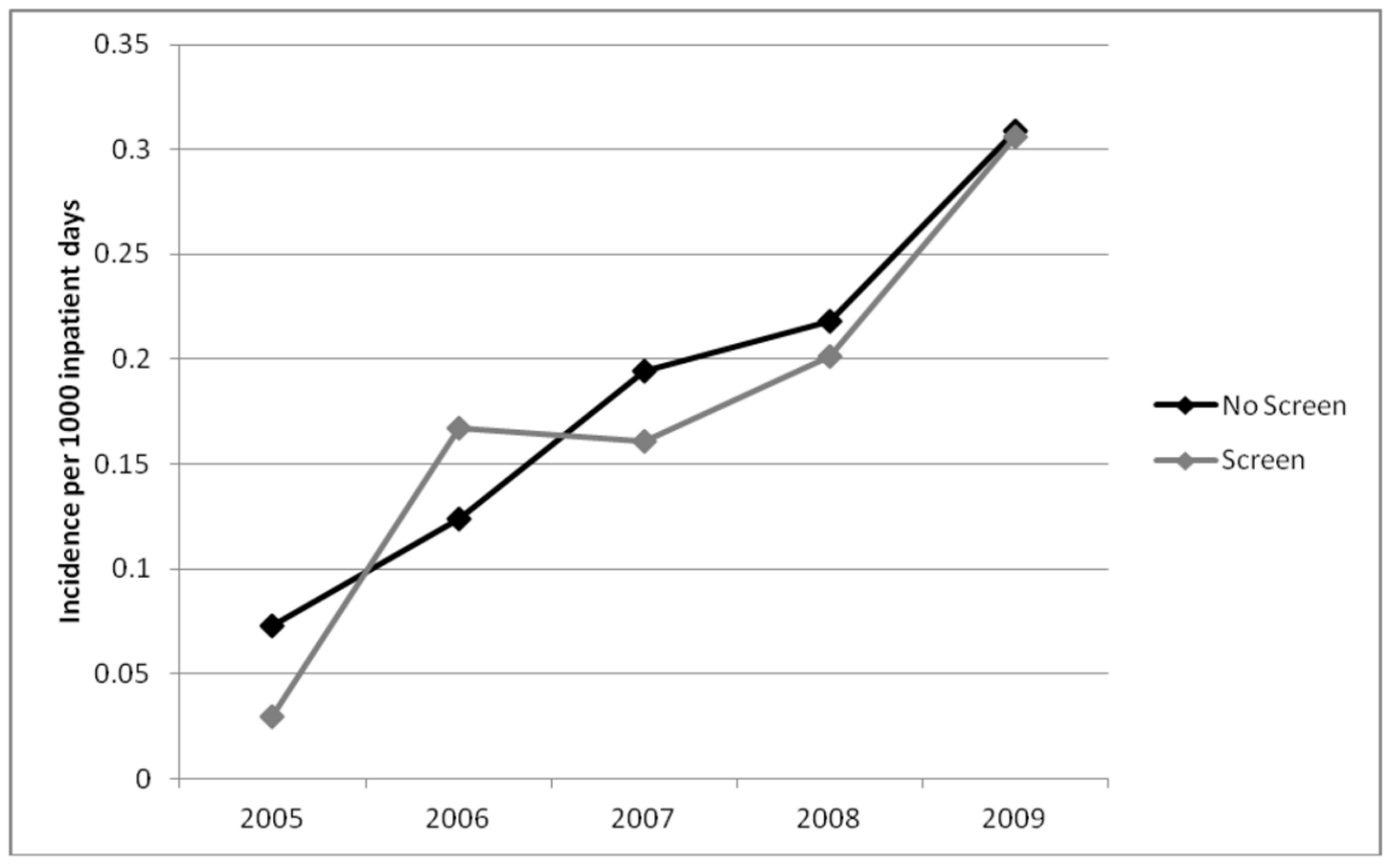
Incidence of community-onset cases of ESBL-producing *Enterobacteriaceae* in non-screening compared to screening hospitals.

**Figure 4:**
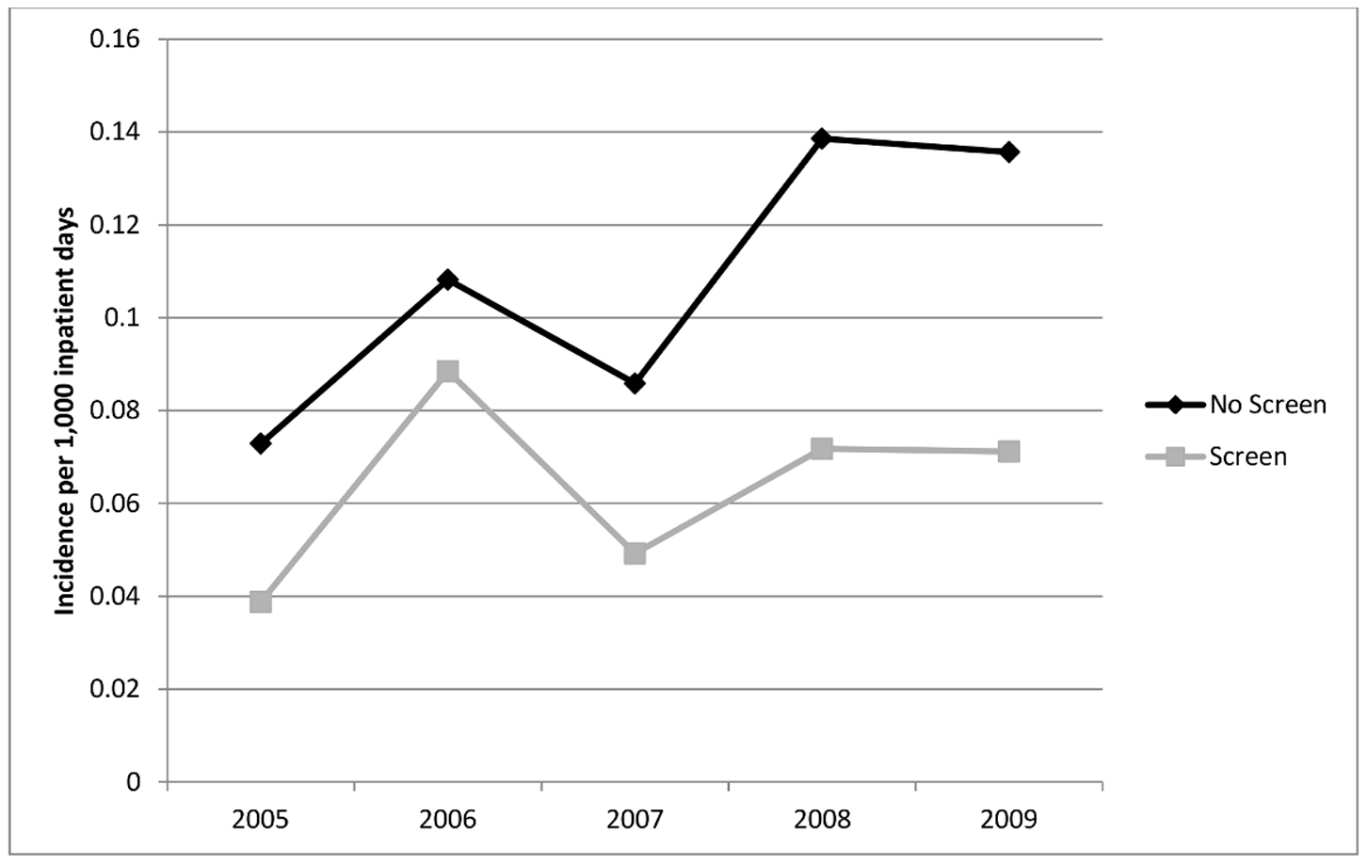
Incidence of hospital-onset cases of ESBL-producing ***Escherichia coli*** **in non-screening compared to screening hospitals.**

**Figure 5:**
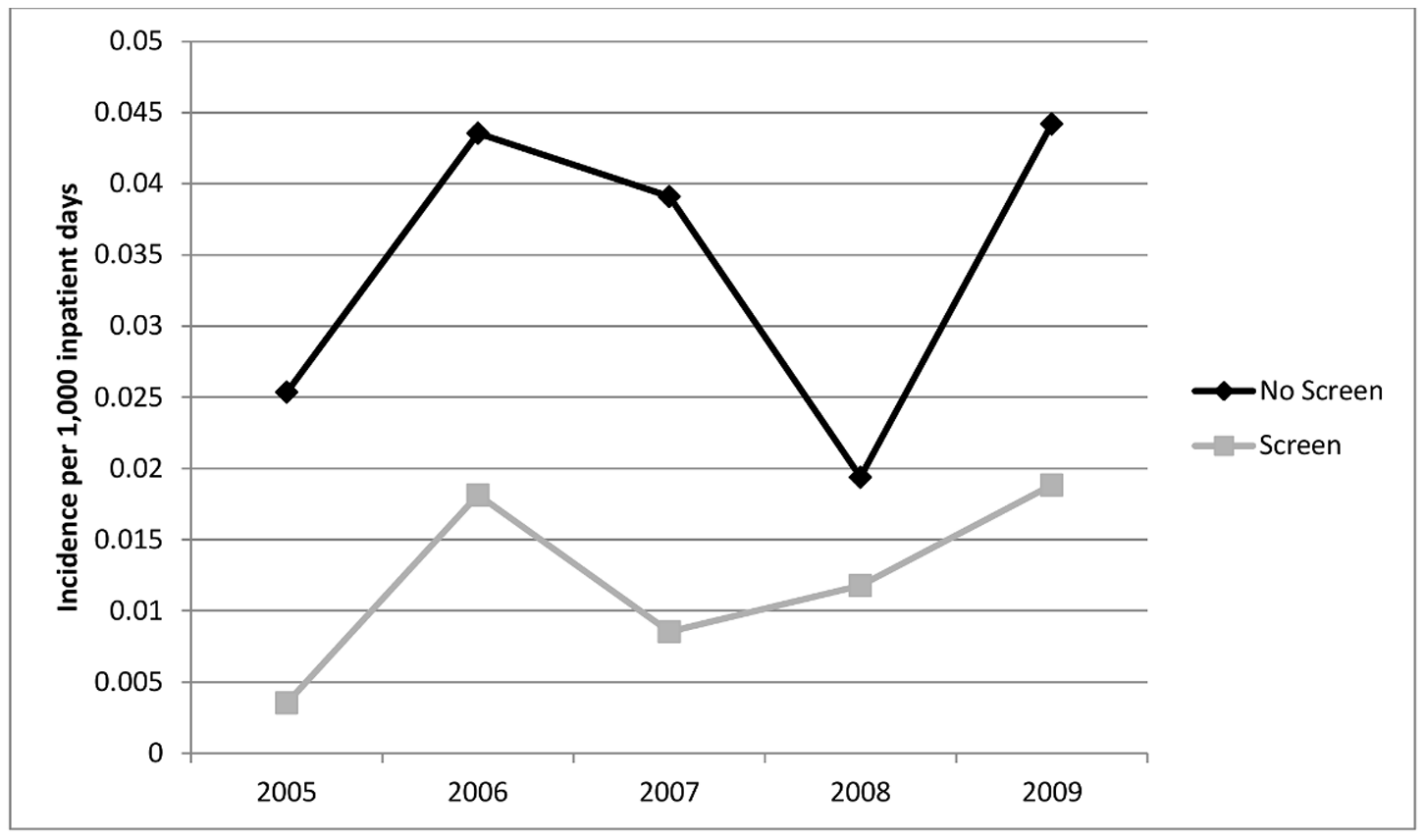
Incidence of hospital-onset cases of ESBL-producing *Klebsiella pneumoniae* in non-screening compared to screening hospitals.

In the first year of the study, the incidence of HO-ESBL-E cases was higher at non-screening hospitals as compared to screening hospitals (0.098 vs. 0.034 per 1,000 inpatient days). This difference was maintained in all study years despite a rise in the overall incidence of HO and CO-ESBL-E (Figure 2 and 3). By 2009, the incidence of hospital-onset ESBL-E in non-screening and screening hospitals was 0.184 vs. 0.097 per 1,000 inpatient days, respectively. The overall crude incidence of HO-ESBL-E for all study years was 641 cases in non-screening and 244 cases in screening hospitals. This difference was seen for both *E. coli* (Figure 4) and *K. pneumoniae* (Figure 5).

In contrast, the rate of community-onset ESBL-E was similar in screening and non-screening institutions throughout the study period (Figure 3). The HO/CO ratio was higher for the non-screening hospitals (0.88 vs. 0.45) and was consistent for both *E. coli* (0.74 vs. 0.39) and *K. pneumoniae* (2.26 vs. 1.76).

**Figure 6:**
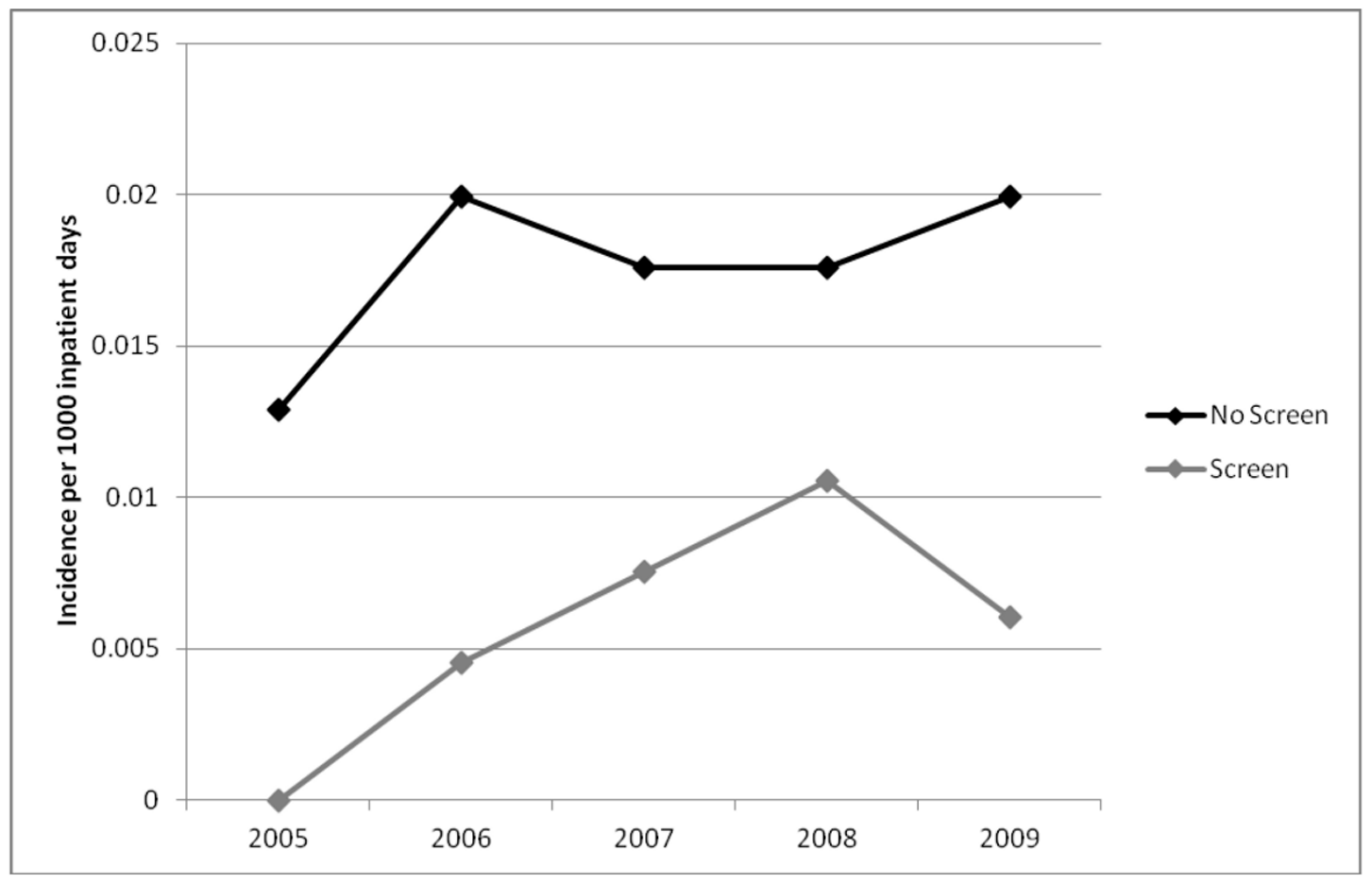
Incidence of hospital-onset ESBL-producing *Enterobacteriaceae* bacteremia in non-screening compared to screening hospitals.

In the negative binomial model, screening hospitals had a 49.1% (parameter estimate = −0.68, p<0.001) reduction in hospital-onset cases compared to non-screening hospitals. This was not associated with the year of specimen collection (estimate = 0.043, p = 0.47), but correlated with increasing community cases (estimate = 2.13, p<0.001). These results were similar when analyzed by organism: *E. coli* (estimate = −0.72, p<0.001) and *K. pneumoniae* (estimate = −0.86, p = 0.001). Analysis of hospital-onset bloodstream infections also demonstrated lower rates for screening hospitals as compared to non-screening hospitals in all study years (Figure 6). In a negative binomial model with only HO-ESBL-E bacteremias included, incidence of HO-ESBL-E bacteremia in screening hospitals was 64.1% lower. The total incidence of bloodstream infections is presented in Table 1.

### Analysis of Screening Hospitals Alone

Incidence of HO-ESBL-E clinical isolates in relation to the total percentage of patients screened is shown in Table 2. There was a trend towards a lower incidence of HO-ESBL-E in hospitals with a higher proportion of admitted patients screened (R4– R6) (P = 0.07, Wilcoxon rank sum). Hospital R2 and R3 had the highest proportion of CO-ESBL-E who did not have an admission screen, and these 2 hospitals were also the only screening hospitals that did not isolate all ESBL-E positive patients. Hospital R1 is an outlier, with the highest rate of capture of patients admitted with a clinical ESBL-E isolate but a relatively low proportion of admissions screened.

**Table 2:** Dose-response relationship of screening intensity and incidence of hospital-onset ESBL-producing *Enterobacteriaceae* in screening hospitals (2009).

Hospital	% of patients screened forESBL-E on admission	% of patients with a clinical ESBL-E isolate within 72 hours of admission without an admission screen	Incidence of HO-ESBL-E clinical isolates per 1,000 inpatient days
R4	87.3	37.5	0.047
R6	62.7	47.1	0.058
R5	73.6	39.3	0.106
R1	41.3	13.3	0.106
R3	26.6	62.5	0.112
R2	47.1	72.3	0.154

Consideration of the results of the admission screens performed at the 6 screening hospitals allowed for an estimate of the accuracy of our classification of cases as HO-ESBL-E and CO-ESBL-E. Among patients with HO-ESBL-E (N = 243), as defined by time from admission to clinical isolate, reclassification based on admission screen status was as follows: 32.5% (79/243) were confirmed nosocomial cases (admission screen negative), 21.0% (51/243) were probable nosocomial cases (no admission screen) and 46.5% (113/243) were colonized at admission (admission screen positive). Thus, almost 50% of patients classified as HO-ESBL-E based on time from admission to first clinical culture were not truly hospital onset cases.

In screening hospitals, 48.8% (235/482) of CO-ESBL-E cases had a positive admission screen, 6.8% (33/482) had a negative admission screen (false negative), and 44.4% (214/482) had no admission screen performed (i.e. a failure of the screening process to identify at risk patients). The hospitals that most frequently omitted screening for patients with an ESBL-E positive clinical specimen obtained within 72 hours of admission (i.e. CO-ESBL-E) were hospitals R2 and R3 (54% and 61% of cases between 2005 - 2009, respectively), both of which relied on a risk factor based approach to screening. The other four hospitals, which included the two universal screening hospitals (R5 and R6) and the other two risk factor based hospitals (R1 and R4) had rates of missed screens ranging from 23% to 36% (between 2005–2009).

The undetected ratio was 72% (*E. coli* = 73.6% and *K. pneumoniae* = 64.4%) and was stable across the 5 year study. For those with a positive admission screen and no clinical isolate within 72 hours of admission, 5.7% (113/2000) subsequently had a clinical isolate a median of 11 days after admission (range 4–137 days). Six patients (0.3%) developed a bloodstream infection after a positive admission ESBL-E screen a median of 10.5 days after admission (range 4–27 days).

Of patients with an ESBL-E positive clinical specimen, there were 79 patients with a confirmed nosocomial isolate (i.e. negative admission screen and subsequent positive clinical isolate identified >72 hours after admission) including 8 bloodstream infections. *E. coli* accounted for 44.3% (35/79) of these confirmed cases with the remaining attributed to *Klebsiella* spp. Positive clinical cultures were identified within this group a median of 27 days after admission (range 4–148 days) while confirmed nosocomial bloodstream infections occurred a median of 26 days after admission (range 4–37 days).

## Discussion

Unlike organisms such as MRSA and VRE, there is almost no direct evidence of the effectiveness of screening as part of a transmission control program for endemic ESBL-E in hospitals [5]. Additionally, there is controversy with regards to the importance of patient-to-patient transmission of ESBL-E [6], [15], [16] and the sensitivity of the screening test [17]. The epidemiology of transmission may also differ between organisms within the family *Enterobacteriaceae,* with *E. coli* more associated with community acquisition and *Klebsiella* spp. more likely a result of hospital transmission [3]. The higher HO/CO ratio for *Klebsiella* spp. compared to *E. coli* suggests this may be the case. However, in-hospital transmission has been demonstrated for both *E. coli* and *K. pneumoniae* [15], [16], and has resulted in outbreaks of ESBL-E in which interventions including active screening appeared to assist in outbreak control [8], [18].

Our study demonstrated an undetected ratio for ESBL-E of 72%, suggesting that a substantial proportion of patients colonized with ESBL-E will not be identified by clinical cultures alone, one of the primary rationales for active screening [19]. Additionally, we demonstrated that the HO/CO ratio in our population was lower for screening hospitals, providing further evidence that identification and isolation of ESBL-E colonized patients may prevent subsequent nosocomial transmission. In the multivariate model testing our primary hypothesis, we identified a significant association between hospitals that implement active admission screening for ESBL-E and lower rates of HO-ESBL-E transmission. Finally, among screening hospitals, there was some evidence that higher proportions of patients screened were associated with lower rates of HO-ESBL-E infection. In our population, the reduction in HO-ESBL-E infection rates associated with screening was estimated to be approximately 50%, was higher for more severe infection (i.e. bacteremia), was lowest in hospitals screening a greater proportion of admitted patients and was likely an underestimate given that almost 50% of HO-ESBL-E cases were likely acquired prior to hospitalization, potentially diluting the effect of screening.

Additional findings of our study included the identification that the screening strategies employed were imperfect, as 44% of patients presenting with CO-ESBL-E did not have screening specimens performed and 6.8% of admission screens were false negative results. More accurate or expanded risk factors for ESBL-E colonization, use of universal screening at all hospitals, and a more accurate screening test for ESBL-E colonization could further improve the efficacy of active screening in identifying patients colonized at admission and potentially reduce the incidence of ESBL-E transmission.

Our study has several limitations. It was a retrospective study and there was incomplete data from one of the screening hospitals for 2005. However, the impact of screening was consistently seen for all 5 years of the study. Our definition of HO and CO-ESBL-E cases was selected because it could be applied consistently to both screening and non-screening hospitals; this definition, though, frequently misclassified cases as hospital-onset when admission screening indicated that the individual was colonized prior to admission. As already discussed, this would tend to bias our results towards the null hypothesis that active screening is ineffective. Thus, our result may have underestimated the impact of screening. There may have also been systematic differences between screening and non-screening hospitals in terms of their baseline infection control practices (e.g. hand hygiene compliance rates, environmental cleaning protocols, etc.) and patient populations. In particular, most screening hospitals were community hospitals, while most non-screening hospitals were academic hospitals. Additionally, the use of private rooms for ESBL-E patients was more common in hospitals that screened, which may have contributed to the difference identified in the study. Similar to ‘bundled’ approaches for other multidrug resistant organisms, differentiating the effects due to screening, private rooms and contact precautions could not be performed. However, if the combination of screening and isolation was responsible for the observed reduction in HO-ESBL-E incidence, this provides evidence that in-hospital transmission of ESBL-E occurs, is preventable with infection control interventions, and that both rapid identification and isolation of ESBL-E positive patients may be needed to minimize transmission.

In summary, our results raise the hypothesis that admission screening, as a component of the infection prevention and control program for ESBL-E that includes patient isolation, contributed to lower rates of hospital-onset ESBL-E cases in Toronto, including bloodstream infections. Realizing the limitations of a retrospective cohort study, this would not serve as sufficient evidence to implement ESBL-E admission screening, but suggests that prospective studies need to be undertaken. The costs of implementation of ESBL-E control programs are substantial: in one Canadian hospital, the costs of screening, additional precautions, and loss of private room revenue was estimated at over $1,000,000 CAD per year [6]. Cost-benefit analyses need to be conducted, taking into account the potential savings from decreased transmission, both from a financial and public health perspective. With the emergence of CRE and the absence of evidence for CRE admission screening [20], our results can provide guidance for CRE since both resistance mechanisms are plasmid mediated and likely to be transmitted similarly. There is an increasingly scarce selection of effective antimicrobials targeting ESBL-E and attention must shift towards the optimization of infection control practices to prevent the spread of multi-drug resistant *Enterobacteriaceae*.
